# Characteristics of the immune microenvironment and their clinical significance in non-small cell lung cancer patients with ALK-rearranged mutation

**DOI:** 10.3389/fimmu.2022.974581

**Published:** 2022-09-08

**Authors:** Bo Zhang, Jingtong Zeng, Hao Zhang, Shuai Zhu, Hanqing Wang, Jinling He, Lingqi Yang, Ning Zhou, Lingling Zu, Xiaohong Xu, Zuoqing Song, Song Xu

**Affiliations:** ^1^ Department of Lung Cancer Surgery, Tianjin Medical University General Hospital, Tianjin, China; ^2^ Tianjin Key Laboratory of Lung Cancer Metastasis and Tumor Microenvironment, Lung Cancer Institute, Tianjin Medical University General Hospital, Tianjin, China; ^3^ Colleges of Nursing, Tianjin Medical University, Tianjin, China

**Keywords:** ALK-rearranged, EGFR mutation, KRAS mutation, tumor microenvironment (TME), NSCLC

## Abstract

**Background:**

Although immune checkpoint inhibitors (ICIs) are one of the most important treatments for advanced-stage non-small-cell lung cancer (NSCLC), NSCLC patients with ALK-rearranged usually don’t obtain a clinical benefit. The reason may be related to the unique tumor microenvironment (TME). We evaluated the characteristics of immune biomarkers of the TME and their prognostic value in ALK-rearranged NSCLC.

**Methods:**

Tumor samples from patients with ALK-rearranged (N = 39) and EGFR- (N = 40)/KRAS- (N = 30) mutated NSCLC were collected. Immunohistochemistry (IHC) was used to assess the expression of 9 tumor immune markers as well as 6 immune markers of tumor-infiltrating cells. To research the TME of ALK-rearranged NSCLC, EGFR/KRAS-positive patients were used as controls. Furthermore, the correlation between the efficacy and prognosis of patients with advanced-stage (IIIC-IV) ALK rearrangements treated with targeted drugs was analyzed in terms of the TME.

**Results:**

The proportion of PD-L1+ tumors was lower in ALK-positive NSCLC than in KRAS-positive NSCLC. Besides, the proportion of T cells expressing TIM-3-CD8+ (15.38%), CTLA4-CD8+ (12.82%), LAG3-CD8+ (33.33%) and PD-1-CD8+ (2.56%) in ALK-positive NSCLC was lower than that in EGFR/KRAS-positive NSCLC. The expression of CD3, CD8 T cells and CD20 B cells was lower in ALK-positive NSCLC than in KRAS-positive NSCLC (p < 0.0001, < 0.005, and < 0.001, respectively). Nevertheless, the level of CD4 helper T cells was higher in ALK-positive NSCLC than in EGFR/KRAS-positive NSCLC (p < 0.0001 and p < 0.05, respectively). The repression of TIM3 was higher in ALK-positive NSCLC than in KRAS-positive NSCLC (p < 0.001). In addition, our data showed that high expression of PD-L1 (HR = 0.177, 95% CI 0.038–0.852, p = 0.027) and CTLA4 (HR = 0.196, 95% CI 0.041–0.947, p = 0.043) was related to lower OS in advanced-stage ALK- rearranged NSCLC patients treated with ALK tyrosine kinase inhibitors (TKIs).

**Conclusions:**

Immunosuppressive status was characteristic of the TME in patients with ALK-positive NSCLC compared with EGFR/KRAS-positive NSCLC. High expression of PD-L1 and CTLA4 was an adverse prognostic factor in advanced-stage ALK-rearranged NSCLC patients treated with ALK-TKIs. Immunotherapy for ALK-rearranged patients requires further exploration and validation by clinical trials.

## Introduction

Cancer-related deaths are most commonly associated with lung cancer, which is a major global health problem. Non-small-cell lung cancer (NSCLC) accounts for approximately 85% of all lung cancers. The prevalence of anaplastic lymphoma kinase (ALK) translocation positivity is 3-5% (1, 2). A previous study showed that ALK tyrosine kinase inhibitors (TKIs), such as crizotinib or alectinib, improved the prognosis of NSCLC with ALK rearrangements (3, 4).

Although targeted therapies are efficient in the context of oncogenic driver mutations, resistance and tumor recurrence inevitably develop (5). Immune checkpoint inhibitors (ICIs) are a new standard of care for blocking the programmed death-1 (PD-1)/programmed death ligand 1 (PD-L1) axis (6–8). Recently, immunotherapies have been considered an extremely promising therapeutic measure for lung cancer and propelled the field of oncotherapy into a new era. PD-L1 expression in tumor cells is associated with improved clinical outcomes of PD-1 pathway blockade in NSCLC patients (9, 10). Previous trials have reported that the maximum response rate of ICI is usually 20% and that the overall survival (OS) benefit was good in unselected NSCLC patients (11–14). Numerous clinical trials have shown that ICI treatment alone or even in combination with chemotherapy results in significantly longer overall survival (OS) or progression-free survival (PFS) in NSCLC patients with or without high PD-L1 expression (15–19). In contrast, a retrospective analysis found that NSCLC patients harboring EGFR mutations or ALK rearrangements are associated with low responses to PD-1/PD-L1 inhibitors (20). A previous study reported that the response of ALK-positive NSCLC patients treated with ICIs was limited (21). Likewise, the Checkmate 012 trial, a multicohort clinical trial, demonstrated that first-line nivolumab monotherapy or in combination with standard therapies showed no meaningful activity in NSCLC with EGFR mutations (22). Furthermore, numeral clinical trials combining ICIs and TKI resulted in remarkable toxicity without a signal of improved activity above that of the TKI (23–25). Furthermore, previous study had shown that ICIs inhibited more effectively the tumor progression in NSCLC patients with KRAS mutations compared with ALK rearrangements (26).

In addition to PD-1 or PD-L1 immune checkpoints, other immune checkpoints are gradually being identified. Cytotoxic T-lymphocyte antigen-4 (CTLA-4) is negative for T-cell activation. Neoadjuvant therapy in combination with CTLA4 and PD-1 inhibitors has significant benefits in NSCLC (27). Similarly, lymphocyte-activation gene 3 (LAG-3) and mucin-domain containing-3 (TIM-3) are expressed in various kinds of immune cells and transmit inhibitory immune signals (28–30). Combining LAG-3 inhibition with PD-1 blockade can enhance antitumor immunity (28). A previous study reported that TIM-3 positivity was significantly associated with worse prognosis in lung adenocarcinoma (31). T-cell immunoglobulin and ITIM domain (TIGIT), expressed on NK cells, CD8+ T cells and so on, inhibits the antitumor immune responses mediated by T cells and NK cells (32). Moreover, tumor microenvironment (TME) is the environment of all kinds of tumour–immune cells interactions. Spatial profiling technologies are powerful tool to analyze immune cell typing, immune activation and therapeutic options (33, 34). Likewise, co-location of immuno-biomarkers can be used as prognostic indicators (35).

To date, it is still unclear why patients with ALK-positive NSCLC do not benefit from ICIs. Therefore, it is of extreme importance to perform this study to research the TME of these NSCLC patients.

## Materials and methods

### Patient cohorts

A total of 103 NSCLC patients from Tianjin Medical University General Hospital and 6 patients from Tianjin Medical University Cancer Institute and Hospital were enrolled in this study by reviewing medical records between 2014 and 2021. Patients were included in our study if the following criteria were met: 1) confirmed non-small-cell lung cancer according to the eighth edition of the TNM classification of the Union for International Cancer Control; and 2) underwent molecular testing of genetic mutations and was found to be EGFR-, KRAS- or ALK-positive. To obtain a more reasonable dataset for this study, patients were also excluded if they received chemotherapy, targeted therapy or radiotherapy before diagnosis. Because the research was a retrospective chart and specimen review, no personally identifiable information was included.

**Figure 1:**
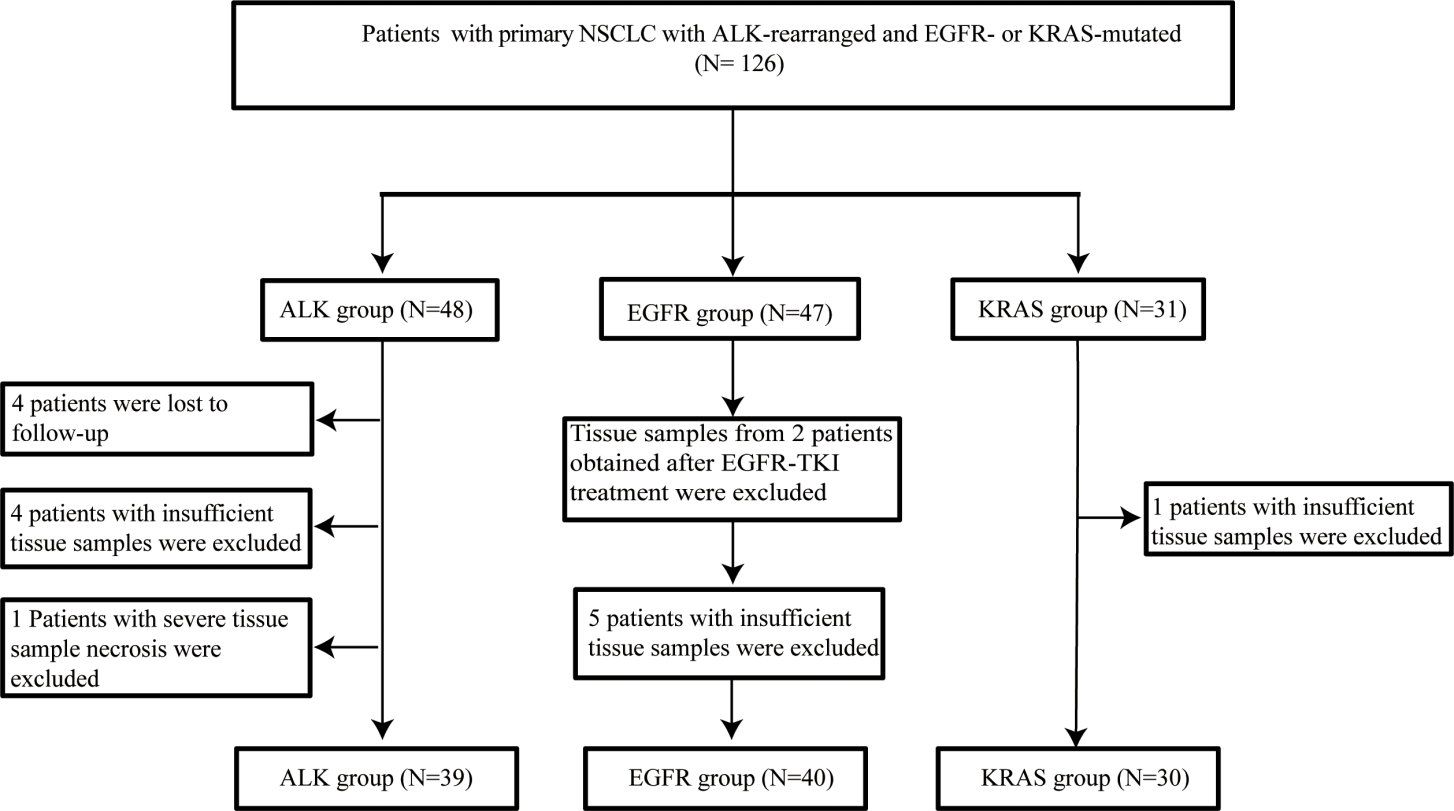
Flow chart for inclusion or exclusion of patients and specimens.

To achieve our objectives, the expression of immune checkpoint molecules (PD-L1, PD-1, LAG3, TIM3, CTLA4, TIGIT, and OX40) and TILs (T cells (CD3+), cytotoxic T cells (CD8+, Granzyme B), macrophages (CD68+), regulatory T cells (FOXP3+, CD4+), NK cells (CD56+), and B lymphocytes (CD20+)) was evaluated by IHC. The flow chart is shown in **Figure 1**. The clinicopathological information of the patients in the study was collected from clinical records and pathology reports. Overall survival (OS) was defined as the time from confirmed diagnosis or treatment initiation to the date of the last follow-up or death due to any cause. If patients had not progressed or died at the time of analysis, they were censored on the date of the last assessment. Survival associations were also assessed for categorical variables using the Kaplan–Meier method, with the log rank test to assess significance. The data were updated as of April 2022. Detailed characteristics of the study cases are presented in **Table 1**. All tissue specimens were used after approval from the Ethics Committee of Tianjin Medical University General Hospital (Ethical No. IRB2021-WZ-055) according to the Declaration of Helsinki. All patients who were enrolled in the study signed informed consent.

**Table 1:** Clinical and pathological characteristics of ALK-/EGFR-/KRAS-positive NSCLC.

Mutation subtype	ALK-rearranged	EGFR-positive	KRAS-positive	P
Number	39	40	30	
Age: (mean ± SD, years)	56.00 ± 11.13	60.05 ± 7.88	65.93 ± 9.22	0.221
< 65	28 (71.79%)	28 (70.00%)	16 (53.33%)	
≥ 65	11 (28.21%)	12 (30.00%)	14 (46.67%)	
Gender				0.081
Male	17 (43.59%)	11 (27.50%)	16 (53.33%)	
Female	22 (56.41%)	29 (72.50%)	14 (46.67%)	
Smoking status:				< 0.001
Current or ever	8 (20.51%)	8 (20.00%)	17 (56.67%)	
Never	31 (79.49%)	32 (80.00%)	13 (43.33%)	
Tumor stage^*^:				< 0.001
I-IIIB	12 (30.77%)	27 (67.50%)	22 (73.33%)	
IIIC-IV	27 (69.23%)	13 (32.50%)	8 (26.67%)	
Histology type				0.531
Adenocarcinoma	38 (97.44%)	40 (100%)	29 (96.67%)	
Non-adenocarcinoma	1 (2.56%)	0 (0%)	1 (3.33%)	
Distant metastasis				0.397
Yes	11 (28.21%)	7 (17.50%)	5 (16.67%)	
No	28 (71.79%)	33 (82.50%)	25 (83.33%)	
ALK TKIs treatment^#^				
Yes	33 (84.62%)	NA	NA	
No	6 (15.38%)	NA	NA	

*AJCC 8^th^ edition.

#ALK TKIs include alectinib or crizotinib.

NA, Not Available.

### Immunohistochemical staining and scoring

The tumor tissues from core biopsy or resected samples were prepared in a tissue microarray format by a professional pathologist. Each of the immunological biomarkers was stained by Immunohistochemical (IHC), respectively, in the continuous pathological sections of formalin-fixed, paraffin-embedded tissue microarray (TMA). The 4 μm TMA was baked at 70°C for 50 minutes, deparaffinized in xylene baths two times for 20 min (Solarbio, China) and rehydrated in graded alcohol baths. Slides were washed three times in phosphate-buffered saline (PBS). Next, antigen retrieval was performed in a microwave oven (Midea, China) with Citrate Antigen Retrieval Solution (Beyotime, China) pH 6.0 for 15 min at 100°C and 10 min at 30°C. The slides were washed as described above. After blocking endogenous peroxidase (ZSGB-BIO, China) for 15 minutes, the slides were incubated with goat serum (ZSGB-BIO, China) for 30 minutes at room temperature. Then, the slides were incubated with the primary antibodies overnight at 4°C in a humidified chamber. Detailed information on the primary antibodies is presented in **Supplementary Table 1**. After washing three times in PBS, the slides were incubated with biotin-labeled goat anti-rabbit IgG polymer (ZSGB-BIO, China) and horseradish peroxidase-conjugated streptavidin (ZSGB-BIO, China) in sequence for 30 minutes and visualized by a 3,3′-diaminobenzidine (DAB) (ZSGB-BIO, China) stain system under a microscope. The slides were counterstained in a hematoxylin dye vat for 30-40 seconds. The slides were washed in PBS and then rehydrated in graded alcohol (70% to 100%) and xylene baths before applying coverslips.

The process of pathological assessment was performed in this way. Firstly, the tumor tissue was assessed and prepared by a professional pathologist, followed by evaluation and scoring with an image-based analysis. Briefly, all stained slides were scanned by a panoramic scanner (Pannoramic MIDI, 3DHISTECH, Hungary) and CaseViewer2.4 software (3DHISTECH, Ltd.). Subsequently, the images were scored automatically by Aipathwell software (Servicebio, Wuhan, China). Finally, quality control of pathological tissues was carried out independent by another professional pathologist. Folds and tears, impurities, stain smudges, tumor necrosis or non-tumor in pathological tissue sections before scanning analysis were all excluded in this study. Some parameters, including positive cells density and the rate of positive cells in the tumor compartment were determined in the tumor compartment (36).

### Statistical analysis

The Kruskal–Wallis U test was used to compare differences between multiple groups. We used the chi-square test to compare differences between categorical variables. Time-dependent receiver operating characteristic (ROC) curves were drawn to identify the optimal cutoff threshold for the high expression of different biomarkers (including PD-L1, PD-1 and CD8). Kaplan–Meier (KM) survival curves and the log-rank test were used to assess the significant differences. The P value was based on a two-sided hypothesis, and a P value < 0.05 was considered statistically significant. ROC analyses and chi-square test were conducted with SPSS 24.0 (SPSS, Chicago, IL, USA). All analyses and survival curves were made with GraphPad Prism (V.8.0.1, La Jolla, CA, USA) and R software (version 4.1.3).

## Results

### Characteristics of immune checkpoint markers in NSCLC with EGFR mutation, KRAS mutation or ALK rearrangement

Among the 109 patients with NSCLC, 39 patients had ALK rearrangements, 40 patients had EGFR mutations, and 30 patients were positive for KRAS mutations. All these patients had tissue slides for PD-L1 staining. Different biomarkers of tissue samples were stained by IHC examination (**Supplementary Figures 1**-**3**).

**Figure 2:**
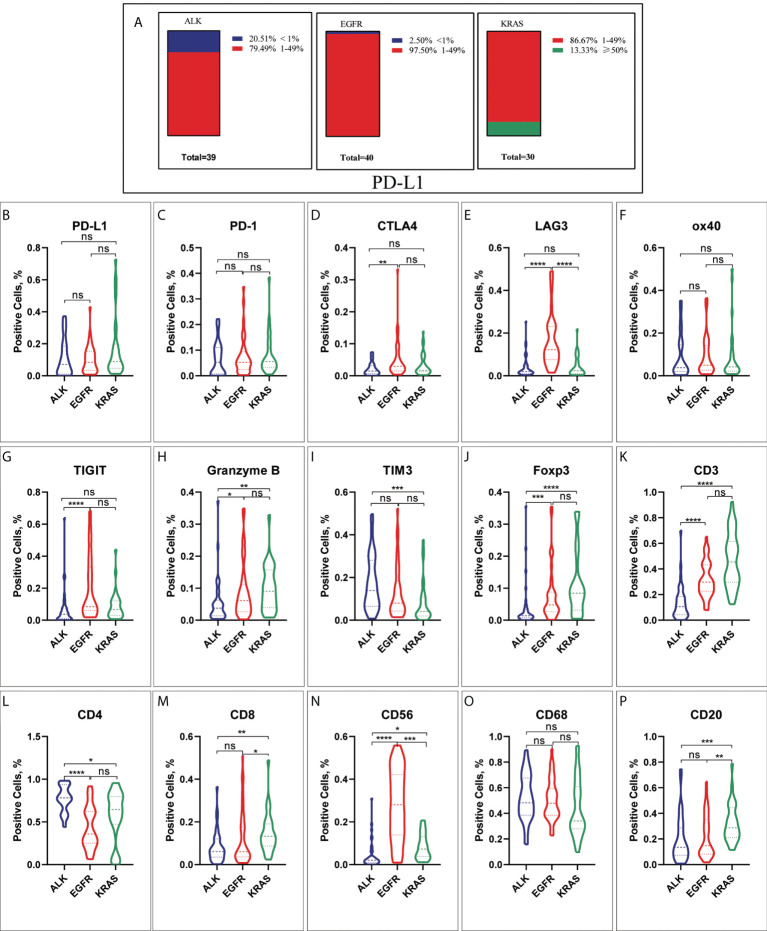
The characteristic of different immune checkpoints in ALK-positive, EGFR-positive and KRAS-positive NSCLC. **(A)** Frequency distribution of PD-L1 expression in ALK-rearranged, EGFR-positive and KRAS-positive NSCLC. **(B)** PD-L1, **(C)** PD-1, **(D)**CTLA4, **(E)** LAG3, **(F)** ox40, **(G)** TIGIT, **(H)** Granzyme B, **(I)** TIM3, **(J)** Foxp3, **(K)** CD3, **(L)** CD4, **(M)** CD8, **(N)** CD56, **(O)** CD68, **(P)** CD20. *p < 0.05, **p < 0.005, ***p < 0.0005, ****p < 0.0001. ns, No significant.

In the ALK-rearranged group, the proportions of PD-L1 in the TME were 0 (≥ 50%), 79.49% (1-49%) and 20.51% (< 1%) (**Figure 2A**). In the EGFR-positive group, the proportions were 0 (≥ 50%), 97.50% (1-49%) and 2.50% (< 1%) (**Figure 2A**). For the KRAS group, the proportions were 13.33% (≥ 50%), 86.67% (1-49%) and 0 (< 1%) (**Figure 2A**). This indicates that the percentage of PD-L1 ≥ 50% in ALK- or EGFR-positive NSCLC patients was lower, and in KRAS-positive NSCLC patients, it was higher. In addition, we compared the expression of PD-L1 in the three groups according to different smoking statuses. We found that the expression of PD-L1 in EGFR-positive patients with a history of smoking was higher than that in patients without a history of smoking (p < 0.05, **Supplement Figure 4**). The expression of PD-L1 in KRAS mutation patients with stage IIIC-IV disease and distant metastasis was higher (all p < 0.05, **Supplemental Figures 5**, **6**).

We investigated nine immune markers (PD-L1, PD-1, TIM3, LAG3, CTLA4, TIGIT, OX40, Granzyme B and Foxp3) and analyzed their expression in TME compartments. Although the expression of PD-1, PD-L1 and OX40 was not significantly different in ALK+, EGFR+ and KRAS+ NSCLC (all p > 0.05, **Figures 2B, C, F**), we discovered that patients with EGFR- and KRAS-positive NSCLC were more likely to have higher expression levels of granzyme B and Foxp3 than patients with ALK-positive NSCLC (all p < 0.05 **Figures 2H, J**). Patients with ALK rearrangements had lower expression levels of CTLA4, LAG3 and TIGIT in the TME than patients with EGFR mutations (all p < 0.05, **Figures 2D, E, G**). In contrast, the data verified that TIM3 expression was significantly increased in patients with ALK-positive NSCLC compared to patients with KRAS-positive NSCLC (p < 0.05, **Figure 2I**).

### Characteristics of immune cell infiltration in the TME

The distribution of different TILs was analyzed in specimens from ALK-, EGFR- or KRAS-positive NSCLC patients by IHC examination. The statistical results showed that the proportions of CD3+, CD8+ and CD20+ TILs were significantly different between the KRAS+ and ALK+ and EGFR+ NSCLC groups, and the proportion was higher in KRAS+ NSCLC patients (all p < 0.05, **Figures 2K, M, P**). Conversely, the expression of CD4+ T cells was higher in the ALK-positive NSCLC group than in the KRAS- and EGFR-positive groups (p < 0.05, **Figure 2L**). As shown in **Figure 2N**, there were more EGFR+ patients with CD56+ NK cells than ALK+ and KRAS+ patients (p < 0.05). Nevertheless, there were no significant differences in CD 68+ macrophages between the ALK+, EGFR+ and KRAS+ NSCLC groups (all p > 0.05, **Figure 2O**).

**Figure 3:**
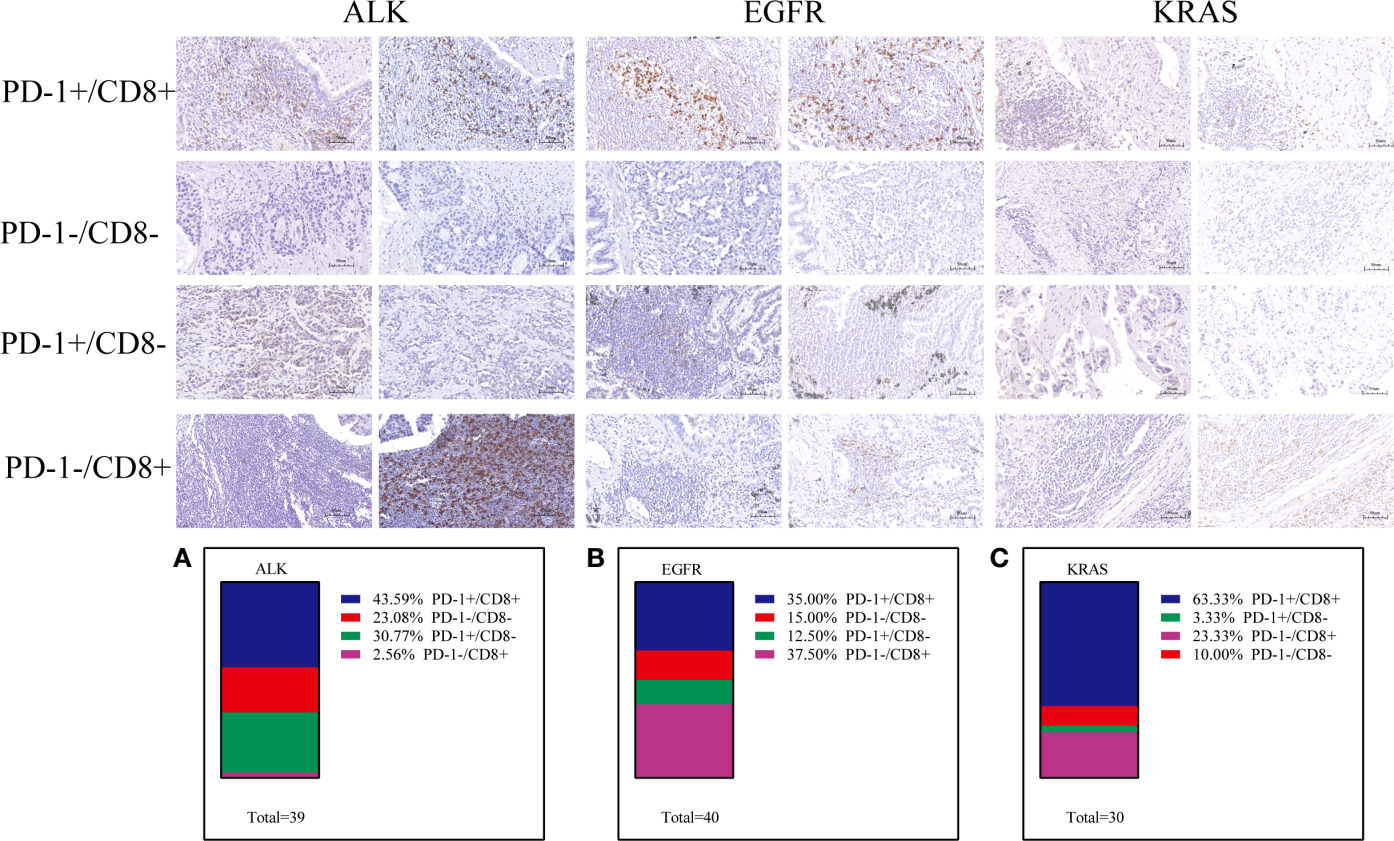
Differential proportion of PD-1+/CD8+, PD-1-/CD8-, PD-1+/CD8-and PD1-/CD8+ in ALK-positive **(A)**, EGFR-positive **(B)** and KRAS-positive **(C)** NSCLC. Low (-) and High (+) expression.

**Figure 4:**
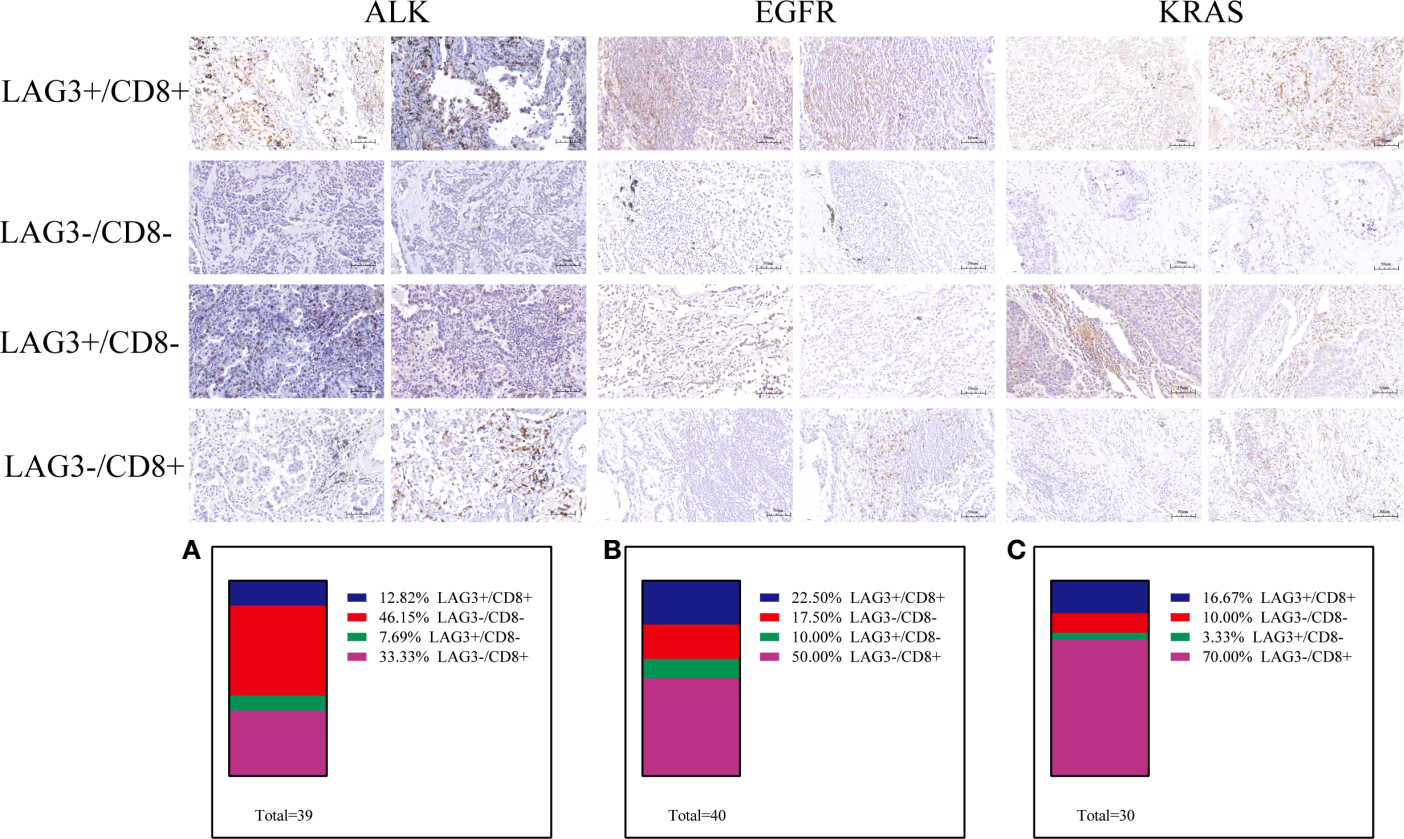
Differential proportion of LAG3+/CD8+, LAG3-/CD8-, LAG3+/CD8-and LAG3-/CD8+ in ALK-positive **(A)**, EGFR-positive **(B)** and KRAS-positive **(C)** NSCLC. Low (-) and High (+) expression.

**Figure 5:**
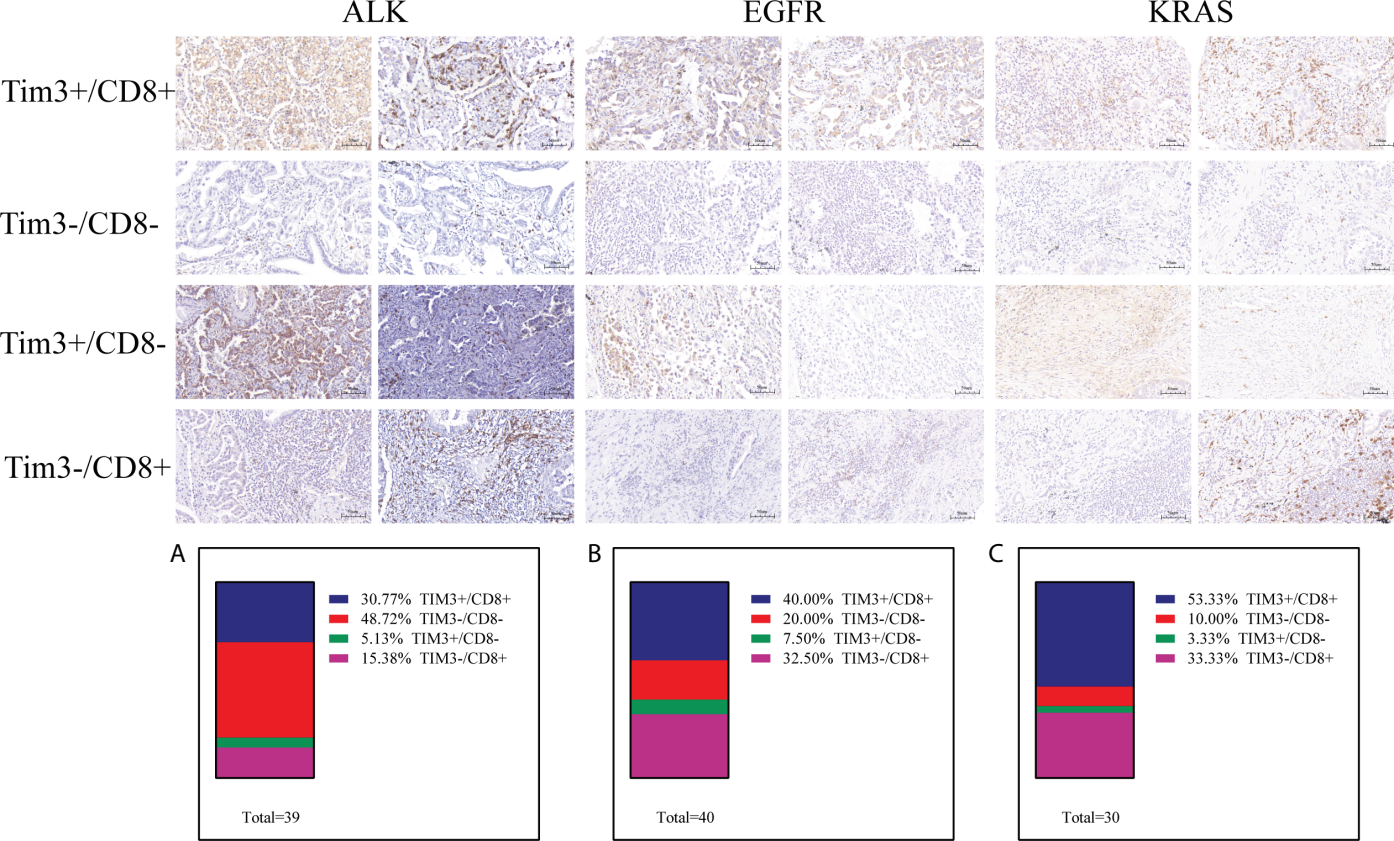
Differential proportion of TIM3+/CD8+, TIM3-/CD8-, TIM3+/CD8-and PTIM3-/CD8+ in ALK-positive **(A)**, EGFR-positive **(B)** and KRAS-positive **(C)** NSCLC. Low (-) and High (+) expression.

**Figure 6:**
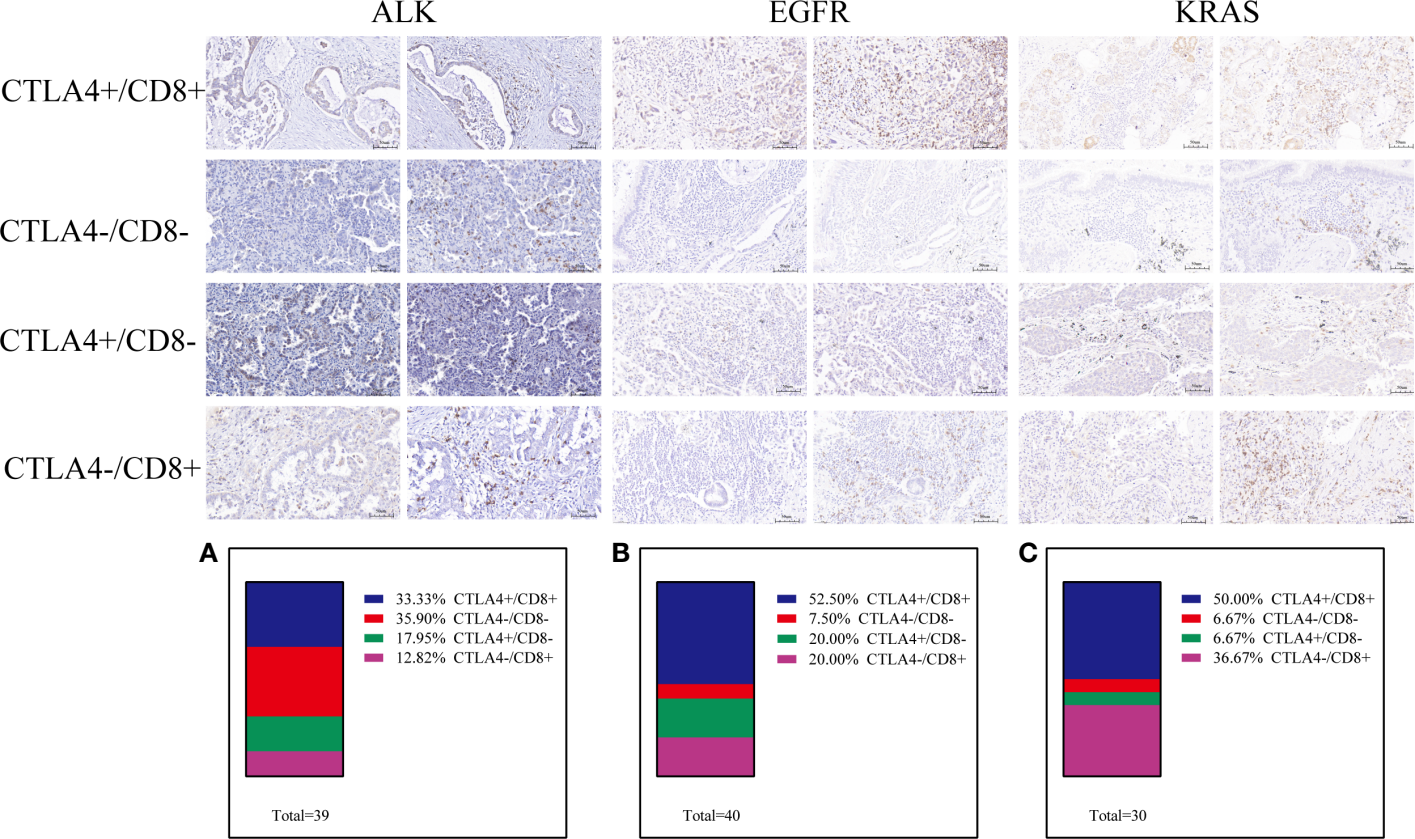
Differential proportion of CTLA4+/CD8+, CTLA4-/CD8-, CTLA4+/CD8- and CTLA4-/CD8+ in ALK-positive **(A)**, EGFR-positive **(B)** and KRAS-positive **(C)** NSCLC. Low (-) and high expression (+).

A previous study defined exhausted T cells as CD8+PD-1+, CD8+LAG3+, CD8+TIM-3+ or CD8+CTLA4+ and non-exhausted T cells as CD8+PD-1–LAG-3–TIM-3– (37–41). Therefore, to explore the function of T cells in the TME, we performed further research. In the ALK-rearranged group, the proportions of PD-1 CD8 T cells in the TME were 43.59% (PD-1+/CD8+), 23.08% (PD-1-/CD8-), 30.77% (PD-1+/CD8-), and 2.56% (PD-1-/CD8+) (**Figure 3A**). In the EGFR+ group, the percentage of exhausted T-cell infiltration in the TME was 35.00% (PD-1+/CD8+), 15.00% (PD-1-/CD8-), 12.50% (PD-1+/CD8-), and 37.50% (PD-1-/CD8+) (**Figure 3B**). In the KRAS-positive group, the proportions were 63.33%, 10.00%, 3.33% and 23.33%, respectively (**Figure 3C**). Likewise, we further researched the distribution of LAG-3 CD8 T cells in the TME. The proportions were 12.82% (LAG-3+/CD8+), 46.15% (LAG-3-/CD8-), 7.69% (LAG-3+/CD8-), and 33.33% (LAG-3-/CD8+) for ALK-positive NSCLC (**Figure 4A**). For EGFR-positive NSCLC, the proportions were 22.50% (LAG-3+/CD8+), 17.50% (LAG-3-/CD8-), 10.00% (LAG-3+/CD8-), and 50.00% (LAG-3-/CD8+) (**Figure 4B**). In the KRAS-positive group, the proportions were 16.67% (LAG-3+/CD8+), 10.00% (LAG-3-/CD8-), 3.33% (LAG-3+/CD8-), and 70.00% (LAG-3-/CD8+) (**Figure 4C**). We next studied the effect of TIM3 CD8 T cells on the three groups of patients and found that the proportions were 30.77% (TIM3+/CD8+), 48.72% (TIM3-/CD8-), 5.13% (TIM3+/CD8-), and 15.38% (TIM3-/CD8+) in the ALK-positive group (**Figure 5A**). For EGFR-positive NSCLC, the proportions were 40.00% (TIM3+/CD8+), 20.00% (TIM3-/CD8-), 7.50% (TIM3+/CD8-), and 32.50% (TIM3-/CD8+) (**Figure 5B**). In the KRAS-positive group, the proportions were 53.33%, 10.00%, 3.33% and 33.33%, respectively (**Figure 5C**). Similarly, the proportions of ALK-rearranged NSCLC were 33.33% (CTLA4+/CD8+), 35.90% (CTLA4-/CD8-), 17.95% (CTLA4+/CD8-), and 12.82% (CTLA4-/CD8+) (**Figure 6A**). For the EGFR group, the proportions of ALK-rearranged NSCLC were 52.50% (CTLA4+/CD8+), 7.50% (CTLA4-/CD8-), 20.00% (CTLA4+/CD8-), and 20.00% (CTLA4-/CD8+) (**Figure 6B**). In the KRAS-positive group, the proportions were 50.00%, 6.67%, 6.67% and 36.67%, respectively (**Figure 6C**).

### Overall survival of ALK-rearranged NSCLC based on TME subtypes

**Figure 7:**
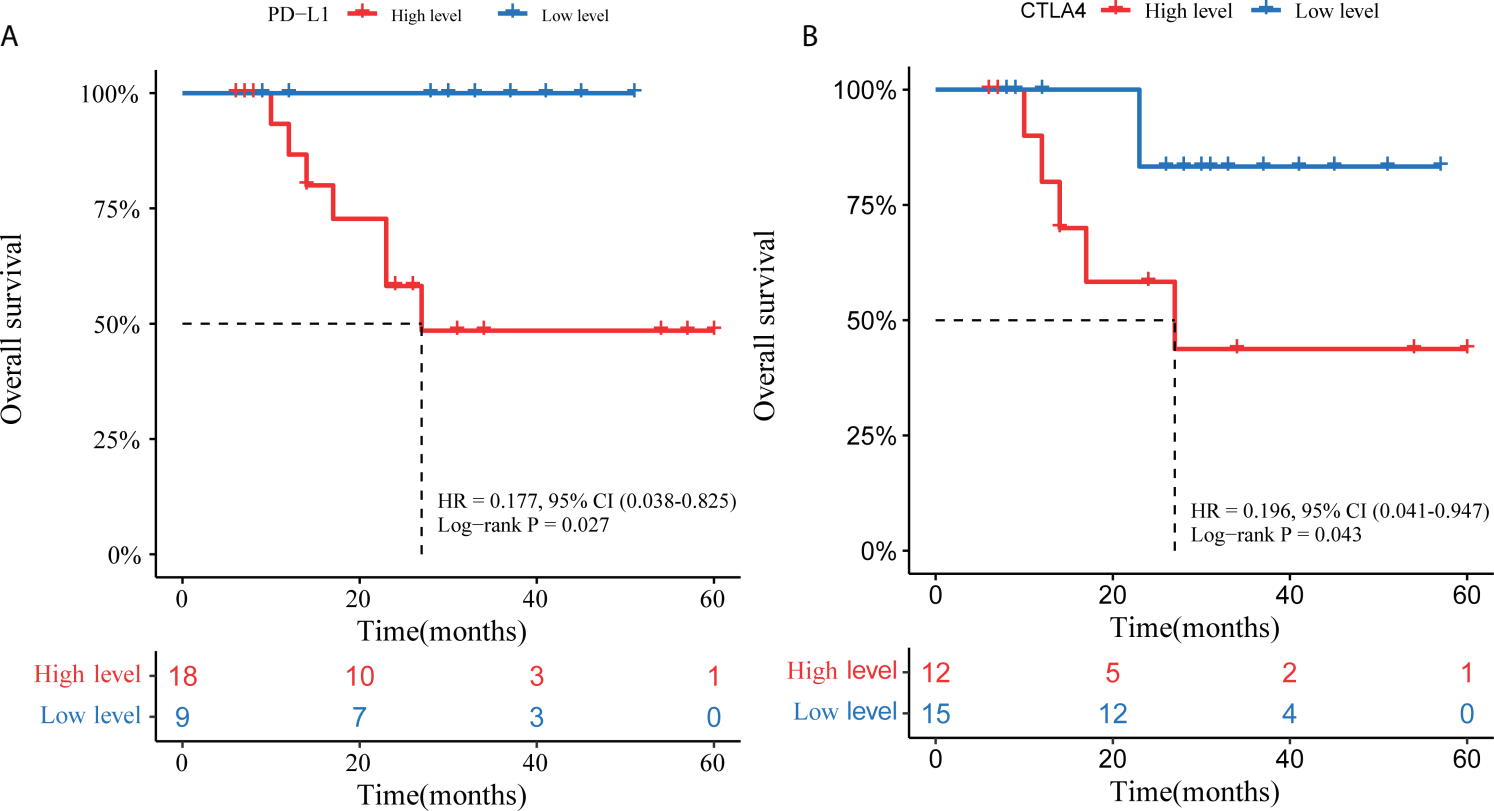
Overall survival Kaplan-Meier curves by PD-L1 **(A)** and CTLA4 **(B)** expression.

To explore the clinical efficacy of TKIs in patients with advanced ALK-positive NSCLC, we performed survival analysis. As shown in **Table 1**, 27 out of 33 ALK-rearranged NSCLC patients who received TKIs were advanced stage (IIIC-IV). The baseline clinical characteristics of these patients are presented in **Supplementary Table 2** and **Supplementary Table 3**. Patients whose tumor samples showed higher PD-L1 expression levels in the tumor cells had a shorter overall survival (OS) than those with lower PD-L1 expression levels (HR = 0.177 [95% CI 0.038–0.825], p = 0.027; **Figure 7A**). Furthermore, we analyzed correlations between the expression of other immunomarkers and OS in the study. The group of patients whose tumor samples showed low CTLA4 expression showed a significant increase in OS compared to the group with high CTLA4 expression (HR = 0.196 [95% CI 0.041–0.947], p = 0.043; **Figure 7B**).

## Discussion

A previous study indicated that NSCLCs harboring ALK rearrangements are associated with low ORRs to PD-1/PD-L1 inhibitors (20), but the specific background of the TME is still unclear. In this regard, we evaluated the different lymphocytic infiltration statuses, expression of immune markers and clinical significance in 109 patients with ALK rearrangements and EGFR/KRAS positivity. This study, to our knowledge, is the largest real-world study which investigates the TME of Chinese patients with ALK rearrangements to date.

Our study found that there was a significant difference in the TMEs of patients with different driver gene positivity statuses. PD-L1≥50% expression was more obvious in KRAS-positive patients than in ALK-/EGFR-positive patients, and the number of PD-L1-positive patients was greater than the number of PD-L1-negative patients in the ALK+ group. A previous study illustrated that high PD-L1 expression was significantly associated with ALK-/EGFR-negative NSCLC (42). Our results showed KRAS-mutant patients had relatively higher rates of concurrent PD-1 expression and CD8+ TILs than ALK-/EGFR-mutant patients. The proportion of non-exhausted CD8+PD-1- T cells in KRAS-mutated NSCLC was higher than that in ALK -mutated NSCLC. Immunological competent cell infiltration was associated with antitumor activity. Preclinical studies have shown that PD-1 inhibitors improve the survival of mice with EGFR-driven lung cancer by enhancing T-cell function (43). Chang Gon Kim et al. showed that patients with PD-1high CD8+ TILs (PD-1-high expressers) exhibited characteristics associated with a favorable anti-PD-1 response compared with those without these lymphocytes (non-PD-1-high expressers) (44). Mechanistically, a low proportion of PD-1+ and CD8+ TILs co-expressed may underlie the low response rates to PD-1/PD-L1 inhibitors in EGFR-/ALK-positive NSCLC patients. Dysfunctional CD8+ TILs expressing inhibitory receptors facilitate tumor immune escape. Our data showed that the proportion of non-exhausted T cells expressing Tim-3-CD8+, CTLA4-CD8+ and PD-1-CD8+ in ALK-positive NSCLC was lower than that in EGFR/KRAS-positive NSCLC. Thus, the TME in patients with ALK-positive NSCLC may be inhibited.

In addition, a study showed that the TME of EGFR-mutant NSCLC was immunosuppressive (45). Previous studies reported that a high proportion of tumor-infiltrating lymphocytes (TILs), particularly those with cytotoxic functions, such as CD8+ and natural killer cells, are correlated with a favorable prognosis in various cancers (46, 47). Our data illustrated that ALK-mutated NSCLC patients had a lower population of activated immune markers, such as CD3, CD8, Granzyme B, and CD20, and a higher expression of immunosuppressive markers, such as TIM3, than KRAS-mutated NSCLC patients. Previous studies showed a similar trend. A previous study showed that TIM3 is a marker of highly suppressive tissue-resident Tregs that play an important role in shaping the antitumor immune response (29). A retrospective analysis showed that NSCLCs with EGFR mutations or ALK rearrangements generally lack T-cell infiltration (20). Nevertheless, in ALK-positive NSCLC patients, the levels of CD4+ helper T cells were significantly higher than those in EGFR-/KRAS-positive patients. Although the TME of ALK-positive patients was different from that of EGFR-/KRAS-positive patients, there was not a total absence of immune infiltration. Our study findings were supported by the work of Jan Budczies et al., who showed that the specific immunosuppressive characteristics of ALK- and EGFR-positive lung adenocarcinoma suggest further clinical evaluation of immune modulators as partners of ICBs in such tumors (48).

To date, no research has performed an overall survival analysis in ALK-positive patients treated with TKI based on PD-L1 expression. Several studies only evaluated the immune landscape with a few markers, such as PD-L1, PD-1, CD3, and CD8 (20, 48, 49). Our results showed that high PD-L1 expression was associated with lower OS than low PD-L1 expression. Cytotoxic T-lymphocyte antigen-4 (CTLA-4) is expressed on the membrane of T cells and inhibits T-cell activation. The immune checkpoint receptor CTLA4 plays a crucial role in negatively regulating function in TME. In our study, in ALK-positive patients treated with TKI, those with low expression of CTLA4 had longer OS, which showed that CTLA4 may have a negative prognostic impact. A clinical trial showed that targeted EGFR or ALK therapy combined with ipilimumab could notably improve PFS and OS (50).

There are still some limitations in our study. First, based on the nature of this retrospective study, some selection biases were inevitable. Second, immunohistochemical assessment based on artificial intelligence is unable to fully distinguish between tumor nests and tumor stroma. Third, we only enrolled patients from a single institution. Other multicenter studies with larger patient cohorts may address these limitations. Lastly, spatial transcriptomics was not performed in this study due to limited tissue specimens.

In summary, the TME of patients with ALK-positive NSCLC was immunosuppressive compared with that of patients with EGFR/KRAS mutations. High expression of PD-L1 and CTLA4 was an adverse prognostic factor in ALK-rearranged NSCLC patients treated with ALK-TKIs. Immunotherapy for ALK-rearranged patients requires further exploration and validation by clinical trials.

## Data availability statement

The original contributions presented in the study are included in the article/**Supplementary Material**. Further inquiries can be directed to the corresponding authors.

## Ethics statement

All tissue specimens were used after approval from the Ethics Committee of Tianjin Medical University General Hospital (Ethical No. IRB2021-WZ-055). The patients/participants provided their written informed consent to participate in this study. Written informed consent was obtained from the individual(s) for the publication of any potentially identifiable images or data included in this article.

## Author contributions

BZ contributed to write the manuscript. BZ, JZ and SZ performed the IHC. BZ, HZ, HW, JH, LY and NZ performed data selection and data analysis. BZ and HZ contributed to the follow-up of patients. LZ and XX contributed to methodology. ZS and SX contributed to the conceptualization, writing—review and editing, supervision and design of the research. All authors contributed to the article and approved the submitted version.

## Funding

The present study was funded by the National Natural Science Foundation of China (82172776), Tianjin Science and Technology Plan Project (19ZXDBSY00060), Tianjin Key Medical Discipline (Specialty) Construction Project (TJYXZDXK-061B), and Diversified Input Project of Tianjin National Natural Science Foundation (21JCYBJC01770).

## Conflict of interest

The authors declare that the research was conducted in the absence of any commercial or financial relationships that could be construed as a potential conflict of interest.

## Publisher’s note

All claims expressed in this article are solely those of the authors and do not necessarily represent those of their affiliated organizations, or those of the publisher, the editors and the reviewers. Any product that may be evaluated in this article, or claim that may be made by its manufacturer, is not guaranteed or endorsed by the publisher.

## Abbreviations

TME, tumor microenvironmen; ALK, anaplastic lymphoma kinase; EGFR, epidermal growth factor receptor; KRAS, kirsten rat sarcoma viral oncogene; ORR, objectives response rates; OS, overall survival; TKIs, tyrosine kinase inhibitor; ICIs, Immune checkpoint inhibitors; TILs, tumor-infiltrating lymphocytes; IHC, immunohistochemistry; SD, standard deviation.
