# Supplementary material for: Characteristics of the immune microenvironment and their clinical significance in non-small cell lung cancer patients with ALK-rearranged mutation
Source: Front Immunol. 2022 Sep 8;13:974581. doi: 10.3389/fimmu.2022.974581 (PMC9494286; doi:10.3389/fimmu.2022.974581)
Supplement: Supplementary file 1 [file Table_1.docx]

Supplementary table 1: Detail information of primary antibody for different biomarkers

| **No.** | **Antibody name** | **Clonality Specie** | **Company** | **Product No.** | **Diluted** | **Identified cells** |
| --- | --- | --- | --- | --- | --- | --- |
| 1 | Anti-TIGIT antibody | Monoclonal Rabbit Anti-human | Cell Signaling Technology | #99567 | 1:500 | CD8+ T cells |
| 2 | Anti-CD8 antibody | Monoclonal Rabbit Anti-human | Cell Signaling Technology | #85336 | 1:300 | CD8+ T cells |
| 3 | Anti-CD3 antibody | Monoclonal Rabbit Anti-human | Cell Signaling Technology | #85061 | 1:300 |  |
| 4 | Anti-CD4 antibody | Monoclonal Rabbit Anti-human | Abcam | ab213215 | 1:50 | CD4+ T cells |
| 5 | Anti-LAG3 antibody | Monoclonal Rabbit Anti-human | Cell Signaling Technology | #15372 | 1:300 |  |
| 6 | Anti-CD68 antibody | Monoclonal Rabbit Anti-human | Cell Signaling Technology | #76437 | 1:500 | macrophages cells |
| 7 | Anti-CD56 antibody | Monoclonal Rabbit Anti-human | Cell Signaling Technology | #99746 | 1:300 | NK cells |
| 8 | Anti-CD20 antibody | Monoclonal Rabbit Anti-human | Cell Signaling Technology | #48750 | 1:300 | B cells |
| 9 | Anti-CTLA4 antibody | Monoclonal Rabbit Anti-human | Cell Signaling Technology | #53560 | 1:300 | regulated T cell |
| 10 | Anti-Granzyme B antibody | Monoclonal Rabbit Anti-human | Cell Signaling Technology | #46890 | 1:100 |  |
| 11 | Anti-OX40 antibody | Monoclonal Rabbit Anti-human | Cell Signaling Technology | #61637 | 1:50 |  |
| 12 | Anti-FoxP3 antibody | Monoclonal Rabbit Anti-human | Cell Signaling Technology | #98377 | 1:100 |  |
| 13 | Anti-PD-L1 antibody | Monoclonal Rabbit Anti-human | Cell Signaling Technology | #13684 | 1:300 | tumor cells |
| 14 | Anti-PD-1 antibody | Monoclonal Rabbit Anti-human | Cell Signaling Technology | #86163 | 1:300 | regulated T cell |
| 15 | Anti-TIM-3 antibody | Monoclonal Rabbit Anti-human | Cell Signaling Technology | #45208 | 1:500 |  |
