# Supplementary material for: Characteristics of the immune microenvironment and their clinical significance in non-small cell lung cancer patients with ALK-rearranged mutation
Source: Front Immunol. 2022 Sep 8;13:974581. doi: 10.3389/fimmu.2022.974581 (PMC9494286; doi:10.3389/fimmu.2022.974581)
Supplement: Supplementary file 2 [file Table_2.docx]

Supplementary Table 2: The baseline clinical characteristics of expression of PD-L1 for patients with ALK rearrangements who received ALK-TKI.

| Expression of PD-L1 | High | Low | P |
| --- | --- | --- | --- |
| Number | 18 | 9 |  |
| Age: (mean ± SD, years) | 58.53±10.52 | 54.25±11.30 | 1.000 |
| < 65 | 10(55.56%) | 5(55.56%) |  |
| ≥ 65 | 8(44.44%) | 4(44.44%) |  |
| Gender |  |  | 0.046 |
| Male | 6(33.33%) | 7(77.78%) |  |
| Female | 12(66.67%) | 2(22.22%) |  |
| Smoking status: |  |  | 0.375 |
| Current or ever | 4(22.22%) | 4(44.44%) |  |
| Never | 14(77.78%) | 5(55.56%) |  |
| Tumor stage^*^: |  |  | 0.636 |
| IIIC | 4(22.22%) | 1(11.11%) |  |
| IV | 14(77.78%) | 8(88.89%) |  |
| Histology type |  |  | 1.000 |
| Adenocarcinoma | 17(94.44%) | 9(100%) |  |
| Non-adenocarcinoma | 1(5.56%) | 0(0%) |  |
| Distant metastasis |  |  | 0.683 |
| Yes | 9(50.00%) | 3(33.33%) |  |
| No | 9(50.00%) | 6(66.67%) |  |

*: AJCC 8^th^ edition
