# Supplementary material for: Characteristics of the immune microenvironment and their clinical significance in non-small cell lung cancer patients with ALK-rearranged mutation
Source: Front Immunol. 2022 Sep 8;13:974581. doi: 10.3389/fimmu.2022.974581 (PMC9494286; doi:10.3389/fimmu.2022.974581)
Supplement: Supplementary file 3 [file Table_3.docx]

Supplementary Table 3: The baseline clinical characteristics of expression of CTLA4 for patients with ALK rearrangements who received ALK-TKI.

| Expression of CTLA4 | High | Low | P |
| --- | --- | --- | --- |
| Number | 12 | 15 |  |
| Age: (mean ± SD,  years) | 57.30±12.24 | 58.00±9.01 | 0.683 |
| < 65 | 9(75.00%) | 9(60.00%) |  |
| ≥ 65 | 3(25.00%) | 6(40.00%) |  |
| Gender |  |  | 0.054 |
| Male | 3(25.00%) | 10(66.67%) |  |
| Female | 9(75.00%) | 5(33.33%) |  |
| Smoking status: |  |  | 0.236 |
| Current or ever | 2(16.67%) | 6(40.00%) |  |
| Never | 10(83.33%) | 9(60.00%) |  |
| Tumor stage^*^: |  |  | 1.000 |
| IIIC | 2(16.67%) | 3(20.00%) |  |
| IV | 10(83.33%) | 12(80.00%) |  |
| Histology type |  |  | 0.444 |
| Adenocarcinoma | 11(91.67%) | 15(100%) |  |
| Non-adenocarcinoma | 1(8.33%) | 0(0%) |  |
| Distant metastasis |  |  | 0.258 |
| Yes | 7(58.33%) | 5(33.33%) |  |
| No | 5(41.67%) | 10(66.67%) |  |

*: AJCC 8^th^ edition
