# Supplementary figures and images for: Characteristics of the immune microenvironment and their clinical significance in non-small cell lung cancer patients with ALK-rearranged mutation
Source: Front Immunol. 2022 Sep 8;13:974581. doi: 10.3389/fimmu.2022.974581 (PMC9494286; doi:10.3389/fimmu.2022.974581)

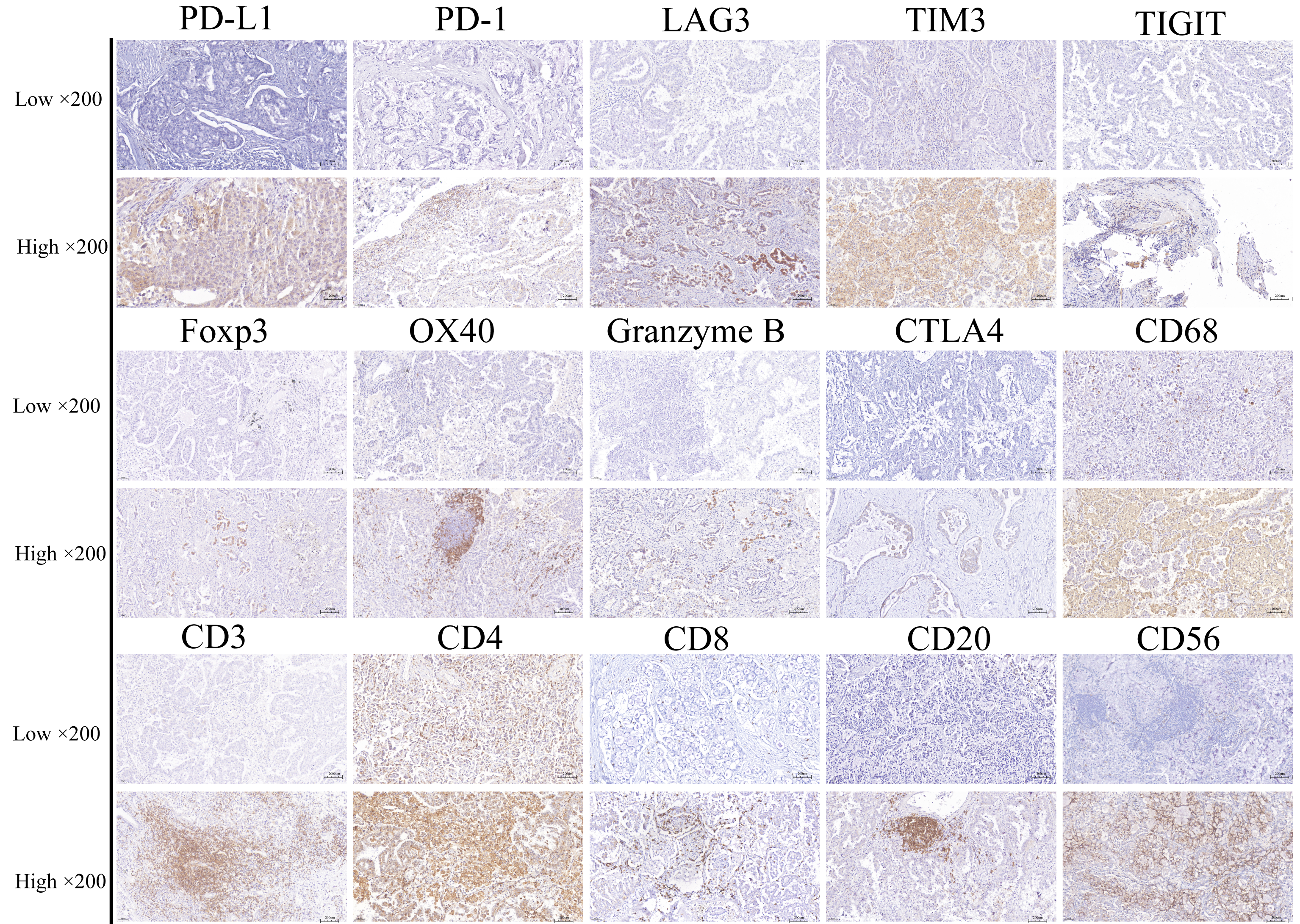

Supplement: Supplementary file 4 [file Image_1.tif]

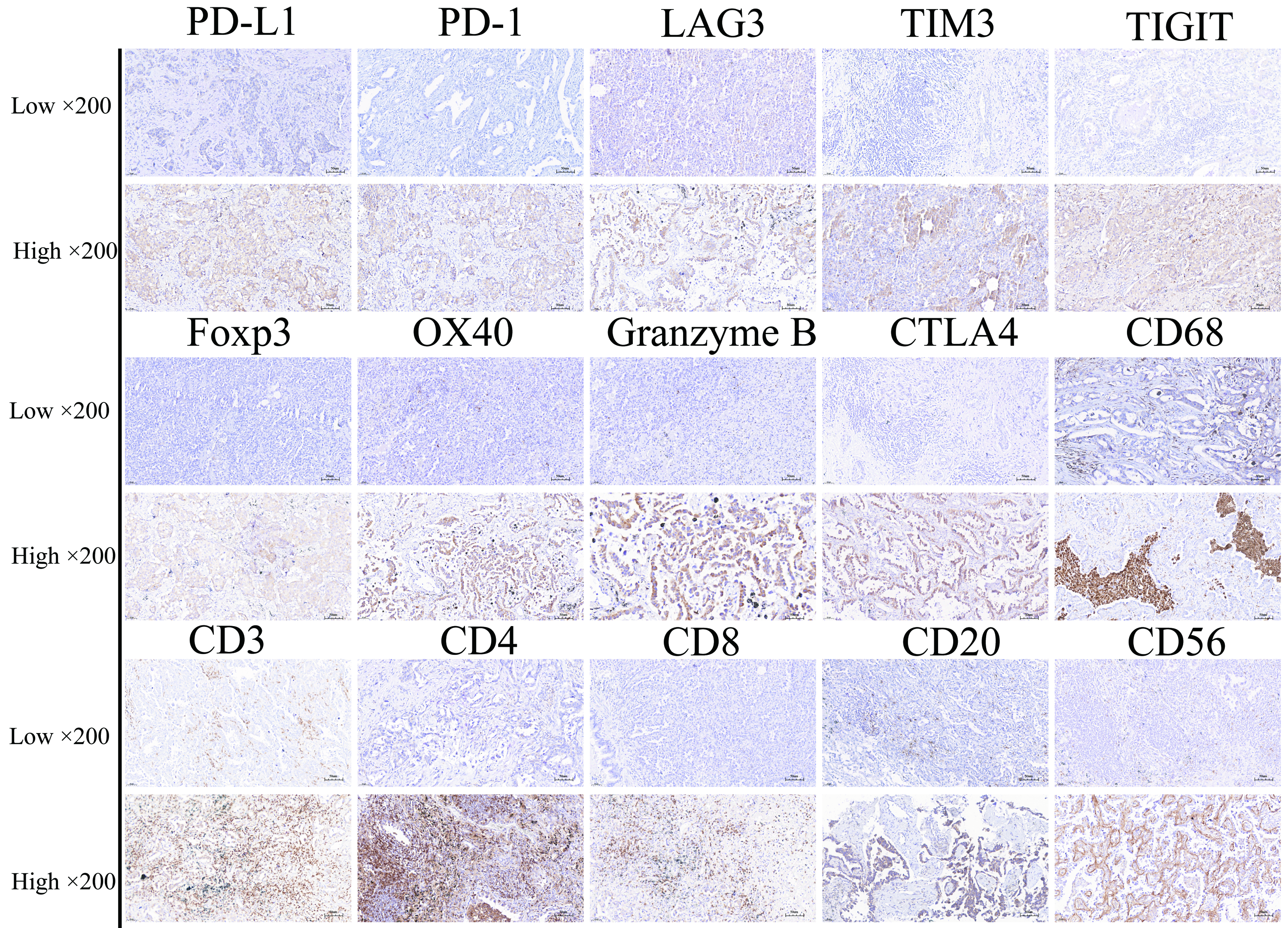

Supplement: Supplementary file 5 [file Image_2.tif]

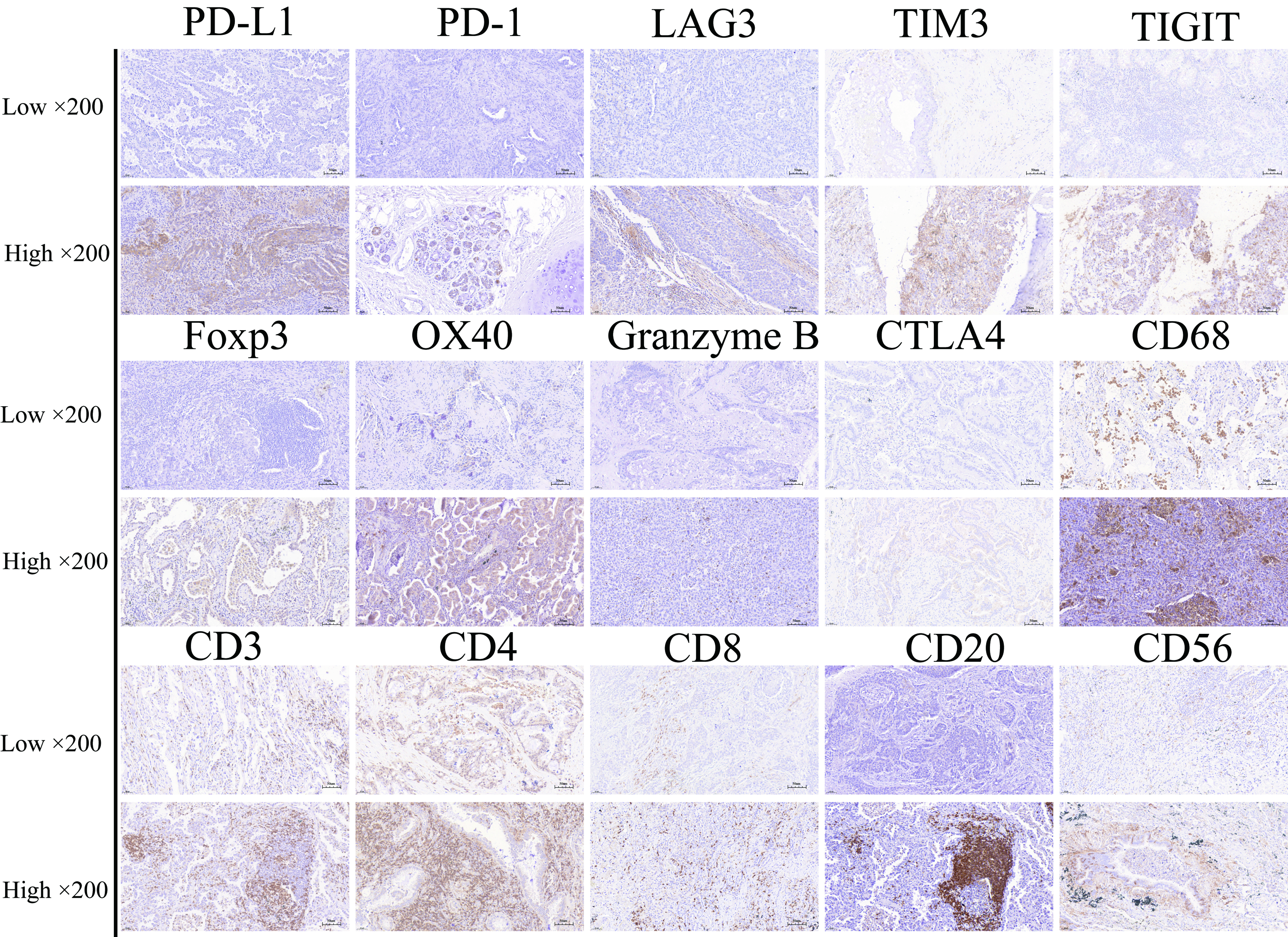

Supplement: Supplementary file 6 [file Image_3.tif]

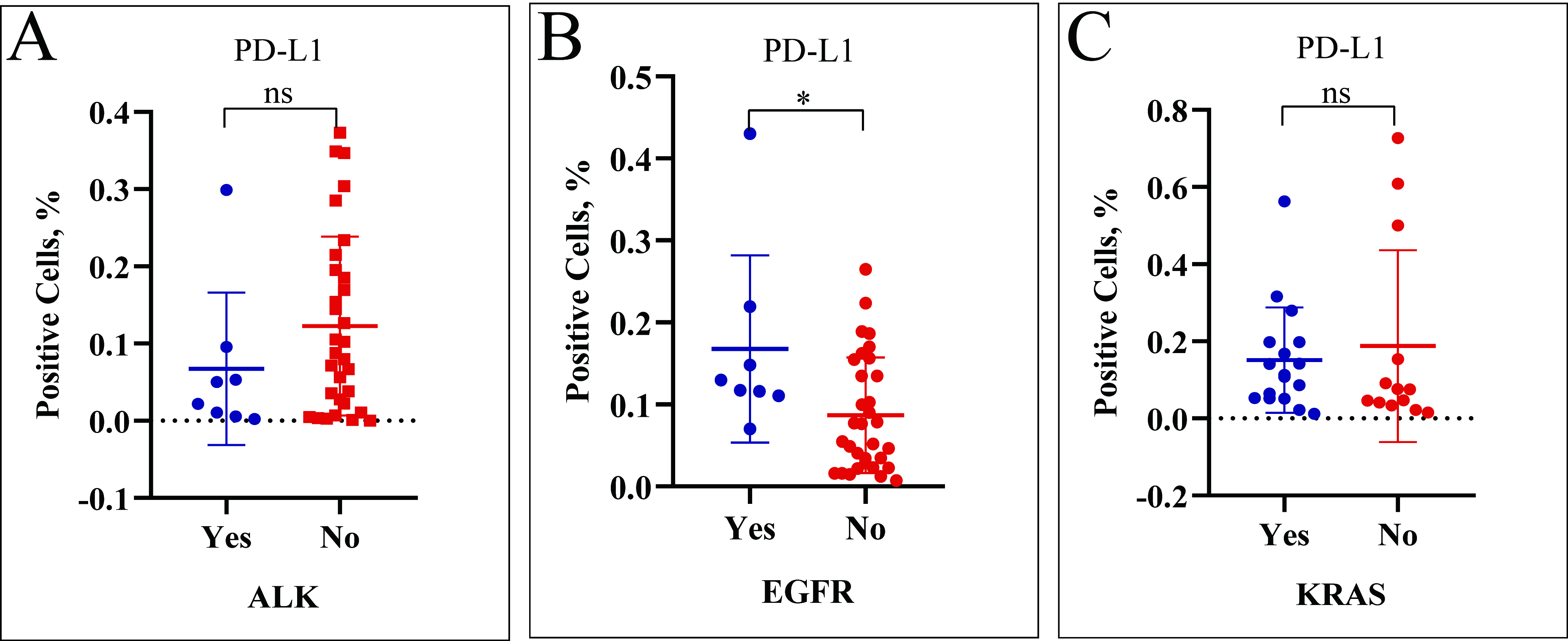

Supplement: Supplementary file 7 [file Image_4.tif]

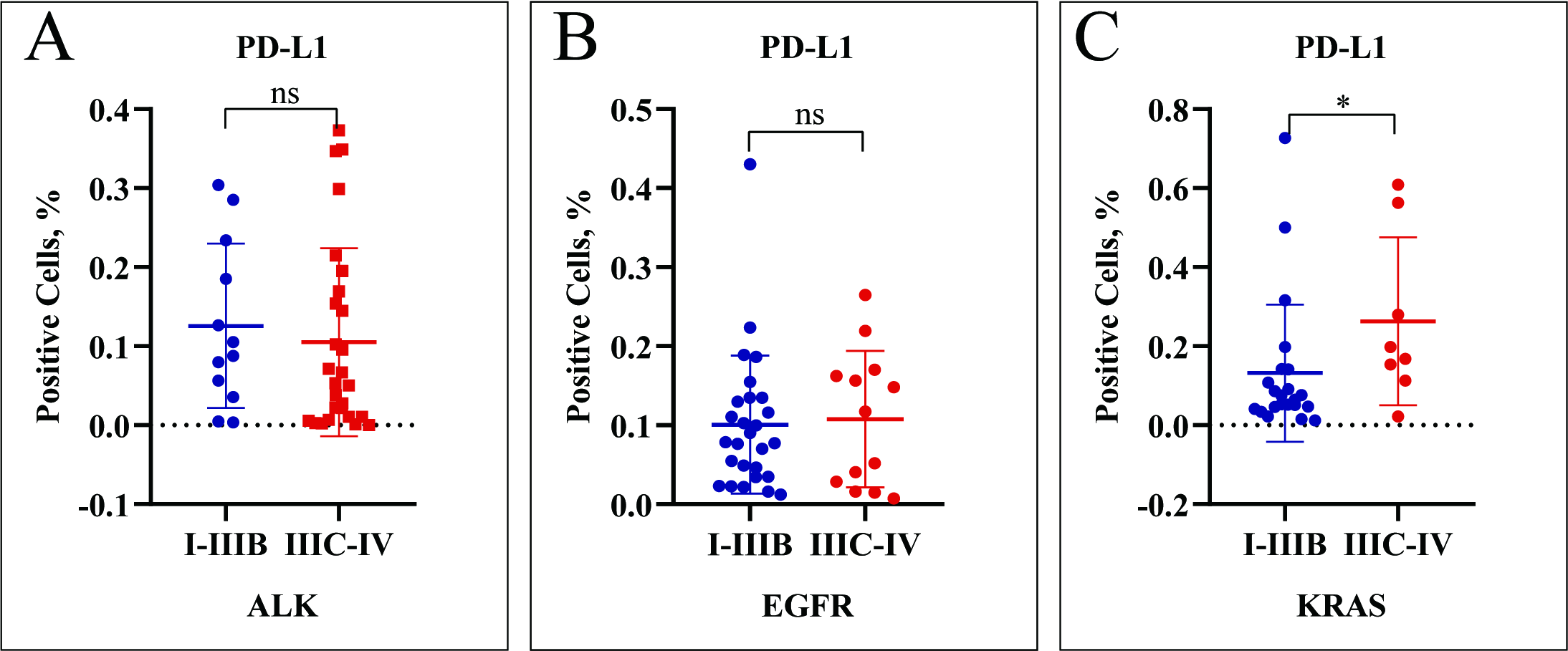

Supplement: Supplementary file 8 [file Image_5.tif]

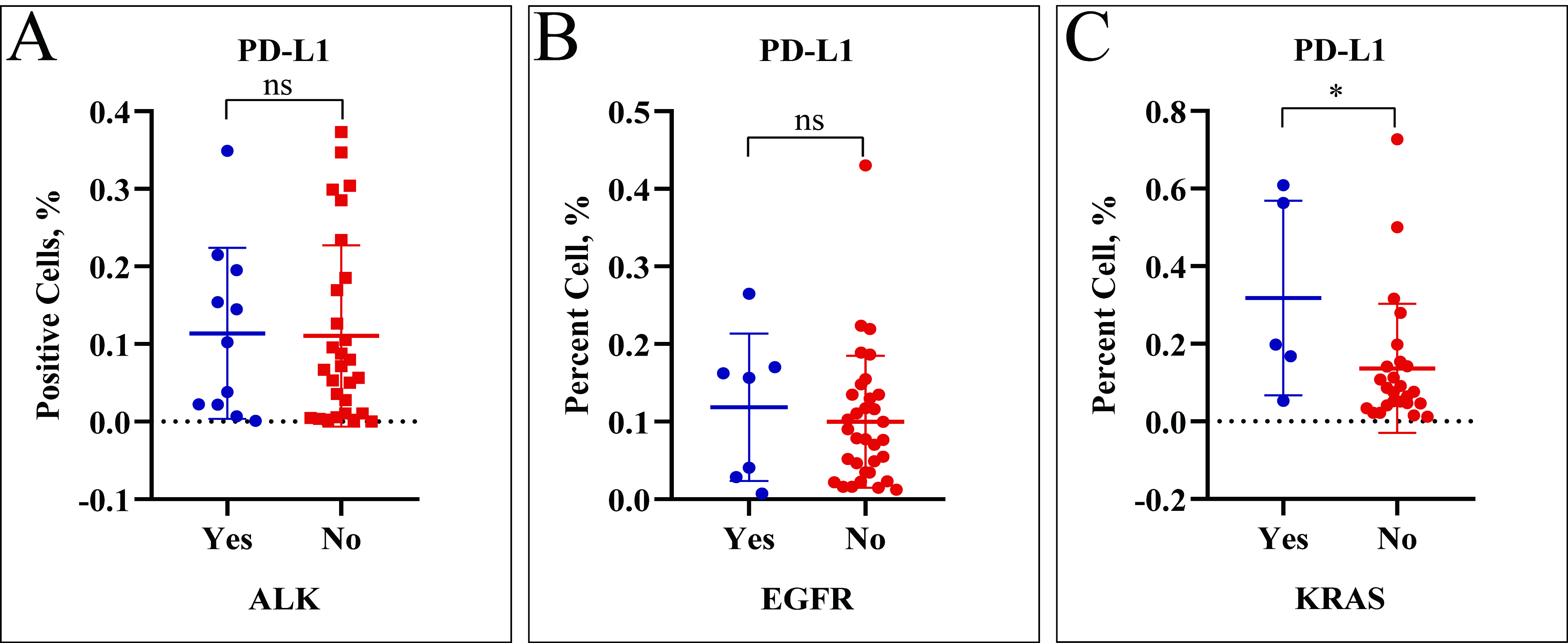

Supplement: Supplementary file 9 [file Image_6.tif]
